# Cutaneous lesions of the nose

**DOI:** 10.1186/1746-160X-6-7

**Published:** 2010-06-04

**Authors:** Michael Sand, Daniel Sand, Christina Thrandorf, Volker Paech, Peter Altmeyer, Falk G Bechara

**Affiliations:** 1 Department of Dermatology and Allergology, Dermatologic Surgery Unit, Ruhr-University Bochum, Gudrunstr. 56, 44791 Bochum, Germany; 2 Case Western Reserve University, School of Medicine, 10900 Euclid Avenue, Cleveland, Ohio 44106, USA

## Abstract

Skin diseases on the nose are seen in a variety of medical disciplines. Dermatologists, otorhinolaryngologists, general practitioners and general plastic and dermatologic surgeons are regularly consulted regarding cutaneous lesions on the nose. This article is the second part of a review series dealing with cutaneous lesions on the head and face, which are frequently seen in daily practice by a dermatologic surgeon. In this review, we focus on those skin diseases on the nose where surgery or laser therapy is considered a possible treatment option or that can be surgically evaluated.

## Review

### Anatomical characteristics

The nose is the central part of the mid-face and has an important functional, aesthetic and psychological role. Nasal respiration, olfaction and phonation are among its most important functional roles. In addition, the aesthetic importance and its impact on the individual psyche have been the subjects of many previous studies [1-3]. For example, when looking at a face, observers spend the largest amount of gaze time on the nose and eyes, underscoring its prominent position in the central face [4].

Because of this exposed, highly visible localization, lesions on the skin of the nose are often noticed by patients themselves, typically very early in the course of the disease. The exposed localization on the face is also cause for increased exposure to ultraviolet (UV) light, which represents one of the most dangerous strains for the skin in this particular location because it is a proven carcinogen. This accounts for the high incidence of cancerous involvement of the skin of the nose, which has proven to be the most common site for skin cancer on the human body [5]. Furthermore, this has lead to the description of the face as a "sun terrace," referring to the skin of the forehead, ears and nose, because the angle of the skin toward sunlight at these locations is more acute than elsewhere. Consequently, UV light exposure is increased, which also includes exposure to the dangerous UV-B spectrum (290-320 nm), shown to be one of the most potent skin carcinogens. Typical UV-B-induced DNA damage involves the generation of dimeric photoproducts between adjacent pyrimidine bases. The tumor suppressor gene p53 is a common target of UV-R-induced mutations. Moreover, UV-A generates highly reactive free radicals, damaging DNA and promoting skin cancer. In addition to its role as a potent carcinogen, UV-A is responsible for damage to the collagen structure, leading to accelerated skin aging [6].

The skin of the nose shows several specific anatomical and histological peculiarities that should be considered when evaluating skin lesions on the nose or when planning the reconstruction of surgical defects [7]. The skin in the areas of the dorsum, columella and sidewalls is thin, loose, compliant and relatively less sebaceous [8,9]. The skin in the areas of the nasal tip and alae is thicker, more sebaceous, more adherent and less flexible [4]. Surgical procedures on the skin of the nose have to respect these different qualities and the nasal topography, including the nasal aesthetic subunits, to achieve the best possible result. The different aesthetic subunits are the tip subunit, columella subunit, dorsal subunit, right and left alar base subunits, right and left alar side wall subunits and right and left dorsal side wall subunits [10]. The anatomical nasal subunits include the dorsum, sidewalls, lobule, soft triangles, alae and columella. The concept of subunits of the external tissue of the nose has proven useful for planning reconstruction. If more than 50% of the subunit is lost it is favorable to replace the whole subunit with regional tissue or a transplant from a donor site [11]. The most important skin diseases on the nose that can require surgical consultation or successfully undergo laser therapy are described below. The description of all dermatoses that can involve the nose would extend beyond the scope of this review. Therefore, our description is limited to those calling for laser or surgical therapy and to those that are clinically most important in the daily practice of a dermatologic surgeon.

## Non-malignant tumors of the nose

A variety of benign skin tumors of the nose are part of daily practice in dermatologic surgery. Such conditions present with different peculiarities and causes. Causes for development of non-malignant tumors of the nose range from simple histomorphologic characteristics, such as the high concentration of sebaceous glands and increased UV-light exposure to more complex genetic abnormalities such as mutations, which can lead to the conditions described briefly below.

### Comedo

**Figure 1:**
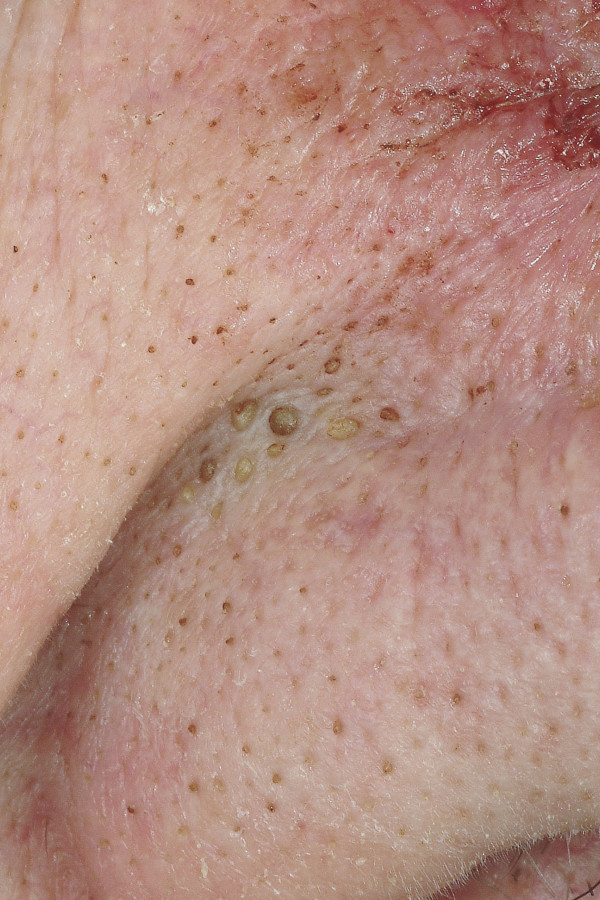
**Comedo**. Multiple closed comedos at the nasolabial fold and the alar of the nose.

Comedos are dilated sebaceous ducts consisting of hyper-proliferating ductal keratinocytes and sebum. They can be either open or closed. The nose with its sebaceous skin at the nasal tip and alae can frequently exhibit comedos [Fig 1]. Interleukin 1-alpha, which is present in 76% of open comedos, induces comedogenesis *in vitro *[12,13]. Furthermore, pilosebaceous ducts have androgen receptors, and estradiol treatment reduces comedos. Therefore, it has been proposed that androgens play a significant role in comedo formation [14,15]. A comedo reaction to different forms of irradiation (megavoltage, cobalt) has been described in the literature [16-20]. Changes in lipid composition of the sebum that lead to duct hyper-proliferation have been hypothesized as causative for this radio-oncologic phenomenon [21]. In addition to desquamation therapy with topical salicylic or retinoic acid, manual extraction by a cosmetician and physical removal by electrocautery or CO_2 _laser therapy have also been reported [22].

### Fibrous papule of the nose (syn.: benign solitary fibrous papule, fibrous papule of the face)

**Figure 2:**
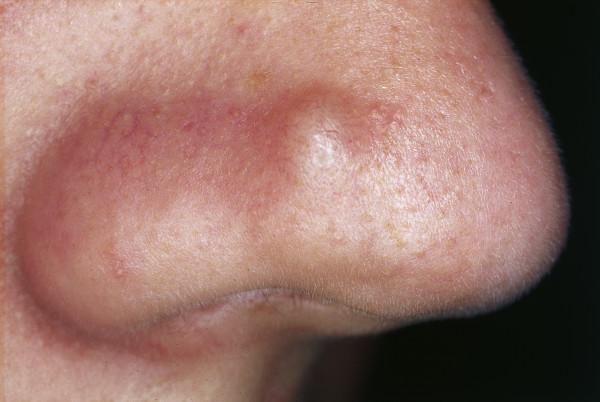
**Fibrous papule of the nose**. Small skin-colored papule with smooth surface.

Fibrous papule is a benign condition that commonly appears on the nose (Fig. 2). The size of the firm papule is between 1-5 mm, and its anatomic distribution predominates at the ala, alar groove and tip of the nose. It has been considered a variant of angiofibroma with a relationship to plasma pro-enzyme factor XIIIa-positive dermal dendrocytes, a population of mononuclear dendritic cells normally present in the papillary and upper reticular dermis [23]. Histopathologically, a clear cell fibrous, hypercellular fibrous, inflammatory fibrous, pigmented fibrous, pleiomorphic fibrous papule and epithelioid variant can be distinguished [24-27]. A biopsy can be necessary to differentiate fibrous papules from benign adnexal tumors or basal cell carcinomas (BCCs) that sometimes closely resemble its "pearly" appearance.

### Adenoma sebaceum (syn.: Pringle disease)

**Figure 3:**
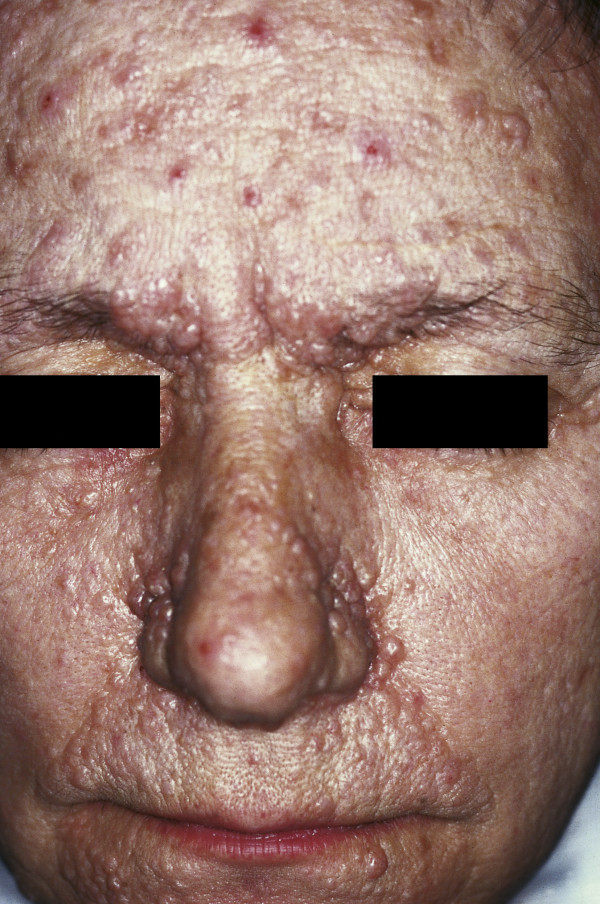
**Adenoma sebaceum**. Multiple wart-like, waxy lumps consisting of angiomatous and fibrous tissue associated with tuberous sclerosis.

Adenoma sebaceum is an archaic misnomer for angiofibromas on the face without any relationship to sebaceous glands. Adenoma sebaceum is part of the classical triad of tuberous sclerosis (adenoma sebaceum, mental retardation and epilepsy), which is an autosomal dominant neurocutaneous disease resulting from the mutation of TSC-1 or TSC-2 [28-30]. The lesions start to occur in childhood (5-10 years of age) and appear as multiple wart-like, waxy lumps consisting of angiomatous and fibrous tissue (Fig. 3). Different therapy modalities such as electrocoagulation, cryosurgery, shave excision and dermabrasion have all been described. CO_2 _laser ablation has been shown to be an effective treatment option, with long-lasting improvement and good cosmetic results [31].

### Hydrocystoma (syn.: cysts of Moll, sudoriferous cysts)

**Figure 4:**
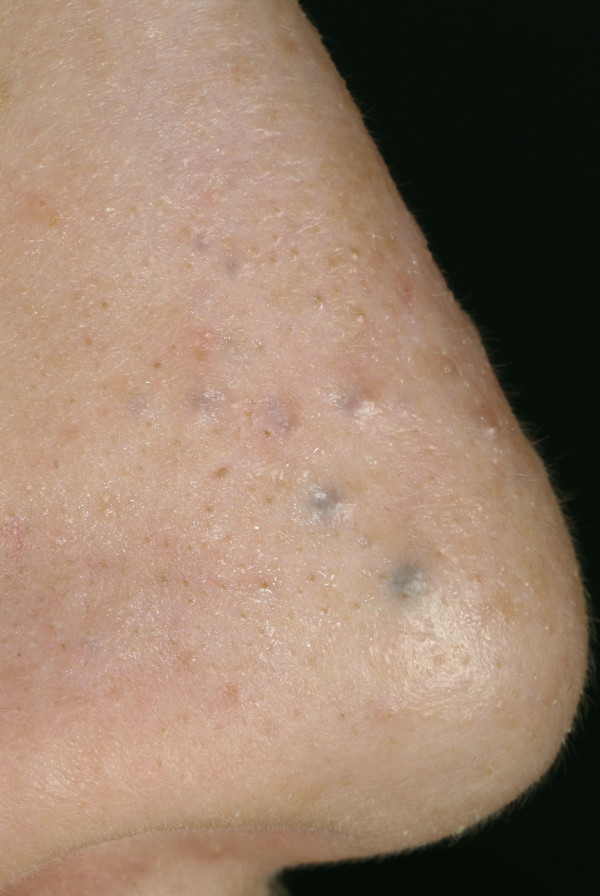
**Eccrine hydrocystoma**. Multiple small papules. Some are skin-colored; the larger papules are dark ("hydrocystome noire").

Hydrocystomas are benign cysts of sweat ducts that arise in the apocrine or eccrine glands (Fig. 4) [32]. They usually present as solitary translucent bluish nodules. The blue color is due to the Tyndall effect, caused by scattered light. Histopathology shows uni- or multilocular cystic spaces within the dermis. Multiple hydrocystomas have been described in Schopf-Schulz-Passarge syndrome, a rare autosomal recessive genodermatosis characterized by palmoplantar keratodermas, eyelid apocrine hydrocystomas, hypodontia, hypotrichosis and onychodystrophy [33]. The treatment of hydrocystomas with topical trichloracetic acid, simple excision, electrosurgery, CO_2 _laser or a 1450-nm diode laser have been described [34-38].

### Sebaceous hyperplasia (syn.: sebaceous gland hyperplasia, senile sebaceous hyperplasia)

Sebaceous hyperplasia is the most frequent benign adnexal tumor displaying sebaceous gland differentiation. Men are more frequently affected than woman. Immunosuppressive therapy (e.g., cyclosporin) can trigger its formation [39]. It is almost always located on the face, including the nose, forehead and lateral cheek parts. Clinically, it appears as a whitish-yellow or skin-colored papule that varies in size (2-6 mm) with often accompanying seborrhoea oleosa and telangiectasias. A central umbilication (from which a small globule of sebum is sometimes expressed) is the most important clinically diagnostic feature for differentiating between BCC and sebaceous hyperplasia [40]. Although it is a completely benign lesion and does not require treatment, it can sometimes be cosmetically disturbing or clinically resemble BCC; therefore, a biopsy might be necessary in some cases. Therapy consists of photodynamic therapy, topical trichloroacetic acid, laser treatment (pulsed-dye or CO_2 _laser), electrosurgery, shave excision, excision or oral isotretinoin therapy for multiple widespread disfiguring sebaceous hyperplasia [41-46].

### Melanocytic papillomatous nevi

Melanocytic papillomatous nevi are acquired dermal nevi that are very common. They protrude from the skin surface and may be pigmented or skin-colored. Upon histological examination, they exhibit nevus cell nests in the dermis. Women are more frequently affected than man (9:1), and the nevi are mostly located on the face [47]. Estrogens might influence the pathogenesis of these distinctive melanocytic nevi [48]. Because the major challenge is to exclude malignancy, histology should not be disregarded in cases of clinical doubt regarding the diagnosis. Therapy consists of excision, shave excision or CO_2 _and erbium: YAG or ruby lasers in cases of a firm clinical diagnosis by an experienced dermatologist.

### Rhinophyma

**Figure 5:**
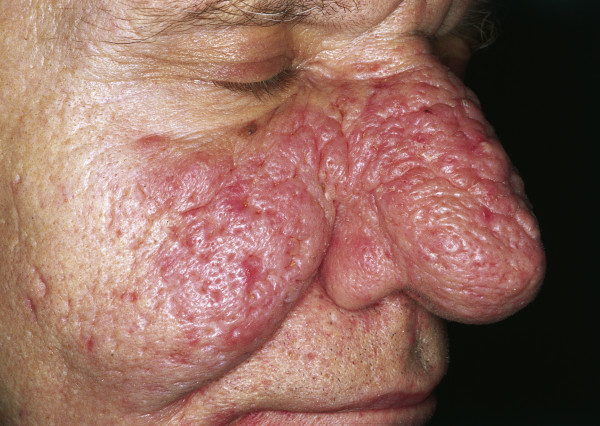
**Rhinophyma**. Large exophytic, pink, lobulated mass over the nose with superficial vascular dilation. The lesion is spreading to the cheeks; however, it can also be limited to the nose.

Rhinophyma is a slow-growing and possibly disfiguring tumor of the nose that primarily affects men in their fifth to seventh decade [49] (Fig. 5). It is characterized by the progressive enlargement of the nose caused by sebaceous hyperplasia, follicular plugging, fibrosis and telangiectasia [50]. Although it is currently classified as stage IV rosacea, some authors believe it represents a different disease process [51]. In the past, rhinophyma has often been associated with heavy alcohol consumption, but new studies have shown that there is no significant correlation [52]. The absence of rosacea skin lesions at adjacent skin areas may be the sign of a tumor mimicking rhinophyma. Although rare, sebaceous carcinomas and angiosarcomas, as well as the more common BCCs and SCCs, are sometimes concomitantly present [53-56]. In rare cases, lupoid cutaneous leishmaniasis can also present as rhinophyma. The removal of excessive tissue can be achieved by dermabrasion, excisional surgery by cold steel, cryosurgery, electrocautery decortication and/or CO_2 _laser ablation [57]. Regardless of the method employed, it is important to respect the delicate anatomy of the nose. The follicular epithelium is the starting point of the re-epithelialization of the wound surface and should not be ablated during rhinophyma surgery [58]. Furthermore, injuries, particularly to the perichondrium of the cartilaginous skeleton of the nose, need to be avoided under all circumstances to prevent nasal flaring.

### Freckles (syn.: Ephelides)

Freckles are small brown macules that are very common, mostly on the face and nose of fair-skinned and red- or blond-haired individuals. They are usually multiple, show no correlation with age and can occur at every age [59]. Histological examination reveals no increase in the concentration of melanocytes. UV light results in larger melanosomes, similar to the melanosomes of dark-skinned individuals [60]. Freckles are not associated with increased mortality but may sometimes represent cosmetic problems for some patients. Therapy consists of sun protection, IPL or Q-switched alexandrite laser treatment [61,62].

## Vascular tumors of the nose

The recent WHO classification of cutaneous vascular tumors differentiates between benign vascular tumors, intermediate vascular tumors, tumors of lymph vessels and tumors of perivascular cells. However, 53 different cutaneous vascular tumors have been described in this classification [63]. Because the face and scalp are common locations, the nose is also often affected by vascular tumors of different origins. The most frequent are described below.

### Hemangioma

**Figure 6:**
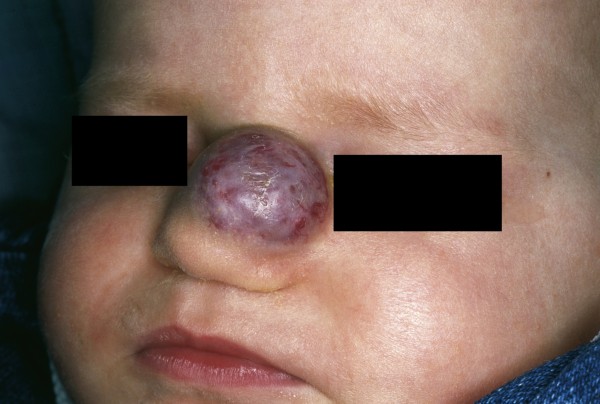
**Infantile hemangioma**. Well-circumscribed red, violet, exophytic vascular tumor on the nose of a one-year-old child.

Hemangiomas are observed in 4-10% of the population and represent the most common tumor of infancy (Fig 6). Caucasians, females (3:1) and premature infants with low birth weight show a higher prevalence [64]. The head and neck are the most common locations (59%) [65]. In facial hemangiomas, 15.8% show involvement of the nose, and the nasal tip is affected in 5.1% [66]. A careful history and examination is the basis for the diagnosis of hemangiomas. Because the lesion is usually absent at birth, it proliferates starting from an erythematous macule or telangiectasia during the first days or weeks of life. The growth phase, which can either be gradual or rapid, is usually six months long and is followed by a longer involution phase of 6-12 months [67,68]. According to Waner et al., facial infantile hemangiomas occur in two distinct patterns of tissue involvement: a focal type with a tumor-like appearance and a less common diffuse type with a plaque-like appearance [69]. The diffuse lesions are more likely to be complicated by ulceration or airway obstruction and show a strikingly segmental distribution pattern compared with focal hemangiomas [66]. Ninety percent of all hemangiomas spontaneously involute prior to the age of 12. Despite this high percentage of spontaneous self-healing, there are still a variety of situations where therapy is indicated. In nasal hemangiomas on the upper third of the nose, the periorbital area is often additionally affected, which can result in impairment of the field of sight. In cases of intraorbital progression, bulbar deviation and amblyopia are dangerous side effects [70]. Nasal involvement can result in nasal deformity (Cyrano nose deformity) or the impairment of nasal breathing [71]. Therefore, treatment of hemangiomas of the nose should be started early to prevent possible complications.

Different therapies such as topical, systemic or intra-lesional applications of steroid, alpha 2a and 2b interferon injections, cytotoxic medications, angiogenesis inhibitors, embolization, cryosurgery, laser therapy and conventional surgery have all been described [72,73]. Imiquimod has also recently been described for the treatment of severe complicated hemangiomas. However, side effects and the small study size make further studies necessary in order to assess this therapeutic option [74]. Recently, Leaute-Labreze and colleagues have achieved impressive results by treating severe fetal hemangiomas of the face with systemic application of the beta-blocker propranolol [75]. After treatment with propranolol administered orally at 2 to 3 mg/kg per day, the authors observed a consistent, rapid, therapeutic effect, leading to a considerable shortening of the natural course of infantile hemangiomas with good clinical tolerance and a low rate of side effects. Initially described in a case report, this has recently been confirmed in larger studies (> 100 patients) [76,77].

### Telangiectasias

Telangiectasias on the nose are extremely common vascular lesions consisting of dilated blood vessels with a linear appearance. They measure between 0.5 and 1 mm in diameter and can be associated with conditions such as rosacea, scleroderma, dermatomyositis, radiation dermatitis, chronic alcoholism, pregnancy, childhood and Osler-Rendu-Weber disease or be idiopathic (as is true in most cases) [78]. When they appear in abundance, telangiectasias on the nose can hint toward heavy liver illnesses or carcinoid syndrome. Although very rare, there are also a group of hereditary telangiectatic syndromes that should be considered when telangiectasias appear in large numbers and during early childhood. These include Rothmund-Thomson syndrome, Bloom syndrome, Cockayne syndrome, ataxia-telangiectasia and hereditary hemorrhagic telangiectasia [79-85]. Former therapy options included needle diathermy occlusion and polidocanol sclerotherapy. However, modern laser treatment has emerged as the first-line therapy for telangiectasias on the face. Good results have been achieved with PDL, long pulsed KTP-Nd: YAG laser and IPL treatment [86,87].

### Spider nevus (syn.: nevus arachnoides, eppinger star, spider angioma, angioma stellatum)

**Figure 7:**
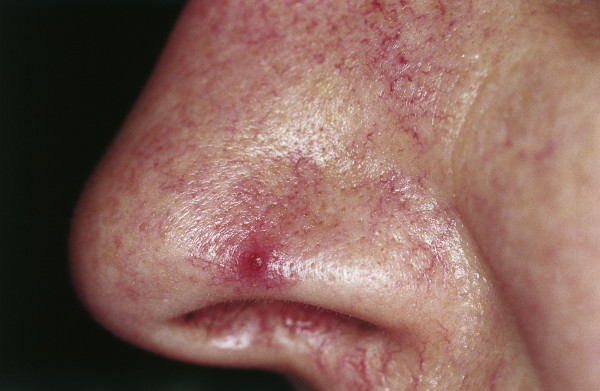
**Nevus araneus (spider nevus)**. In the center of the red lesion a small (1 mm) red papule is visible, surrounded by several distinct radiating vessels. Pressure on the lesion causes it to disappear. Blanching is replaced by rapid refill from the central arteriole when pressure is released.

Spider nevi show a spider-like growth pattern with a pin head-sized central arterial vascular nodule and small vascular radiations in a starburst-like pattern (Fig. 7). When they appear in abundance, spider nevi can be a clinical sign of heavy liver illness or carcinoid syndrome. The most frequent localization is the face and upper body. Under light compression with a glass spatula, arterial pulsations can be recognized in the center, fading toward the periphery. Therapy consists of laser therapy with pulsed dye or alternatively with KTP-Nd: YAG laser or an IPL system [88].

### Osler-Weber-Rendu disease (syn.: hereditary hemorrhagic telangiectasia (HHT))

**Figure 8:**
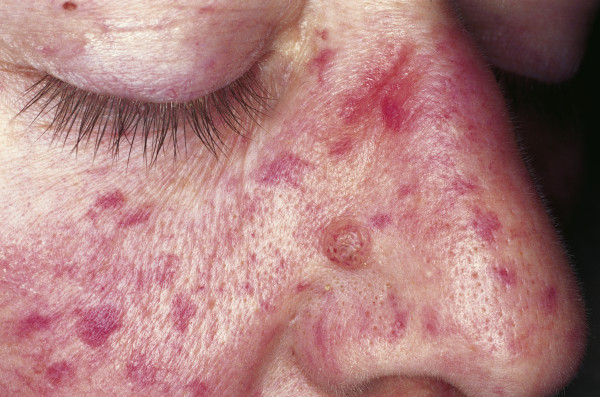
**Hereditary hemorrhagic telangiectasia (Osler-Weber-Rendu syndrome)**. Flat, star-shaped skin lesions 1-3 mm in diameter on the entire face. Some non-pulsating telangiectasias appear similar to araneus nevi. A papule the size of a match head is visible at the alar.

Osler-Weber-Rendu disease is an autosomal dominant disorder that induces the formation of multiple punctate telangiectasias and hemangiomas (Fig. 8). Accompanying epistaxis and mucocutaneous visceral arteriovenous malformations with melena are common. The prevalence is 1-2 per 100,000. Skin lesions can be treated with a long-pulsed Nd: YAG laser, flash-pumped dye laser or an IPL system. Notably, estrogen therapy has been effective in severe cases of Osler-Weber-Rendu disease [89]. Electrocautery or argon beam ablation is described as a possible treatment option for cases of spontaneous recurrent epistaxis [90].

## Inflammatory conditions

The following paragraph describes the most frequent inflammatory conditions on the nose.

### Rosacea

**Figure 9:**
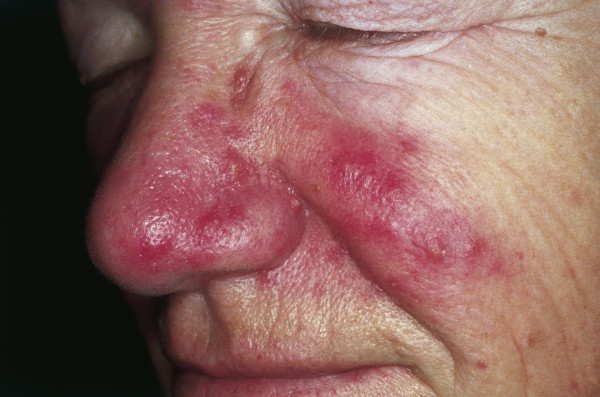
**Rosacea**. Erythema and telangiectasia are seen over the cheeks, nasolabial area and nose. Inflammatory papules and pustules can be observed over the nose. The absence of comedos is a helpful tool to distinguish rosacea from acne.

Rosacea is a multiphasic inflammatory condition that typically affects the skin of the face and nose. Clinically, rosacea has been classified in four different stages. Stage I, also called rosacea erythematosa telangiectasia (pre-rosacea), shows facial flushing and telangiectasia. Stage II, rosacea papulopustulosa (vascular rosacea), is characterized by persistent facial erythema, telangiectasia, thickened skin, papules and pustules (Fig 9). Stage III, glandular-hypertrophic or inflammatory rosacea, shows erythematous papules and pustules, telangiectasias, edema, connective tissue and sebaceous gland hyperplasia. Stage IV, or rhinophyma, shows dermal and sebaceous gland hyperplasia, and dilated and cystic sebaceous glands. Most individuals affected by rosacea are of northern European origin, and up to one-third have a family history of the disorder [91]. Clinical signs include facial flushing, erythema, telangiectasia and papulopustular efflorescence similar to acne as described previously. Women are three times more likely to be affected than men, with the reported prevalence between 0.5 and 10% [92,93]. The pathophysiology has been poorly understood, and there have been only limited descriptions of factors that exacerbate or improve this disease [94]. Recent molecular studies suggest that an altered innate immune response is involved in the pathogenesis of vascular and inflammatory disease and is responsible for the observed clinical findings in patients with rosacea [95].

A variety of topical, systemic and physical treatment options are available that have been adjusted to the stage and severity of the disease [96]. Standard topical therapy includes metronidazole 0.75% or 1% gel. Alternatively, azelaic acid 15% gel or 20% cream has also been successfully used in five randomized and controlled studies with good results [97]. Systemic therapy with doxycycline, minocycline, clarithromycin, and moderately high doses of prednisolone or oral isotretinoin has also been described. Persistent erythema and telangiectasia might respond to pulsed dye laser (PDL) and intense pulsed light (IPL) treatments [98]. Furthermore, it is important to remember that ocular rosacea is a potentially blinding eye disorder common in patients with rosacea (6-18% of rosacea patients) [99]. The main symptom is conjunctival injection, which is sometimes accompanied by chalazion or episcleritis. Rosacea patients should therefore be seen by an ophthalmologist early in the disease course [100].

### Facial eosinophilic granuloma (syn.: granuloma faciale, granuloma eosinophilicum faciale)

**Figure 10:**
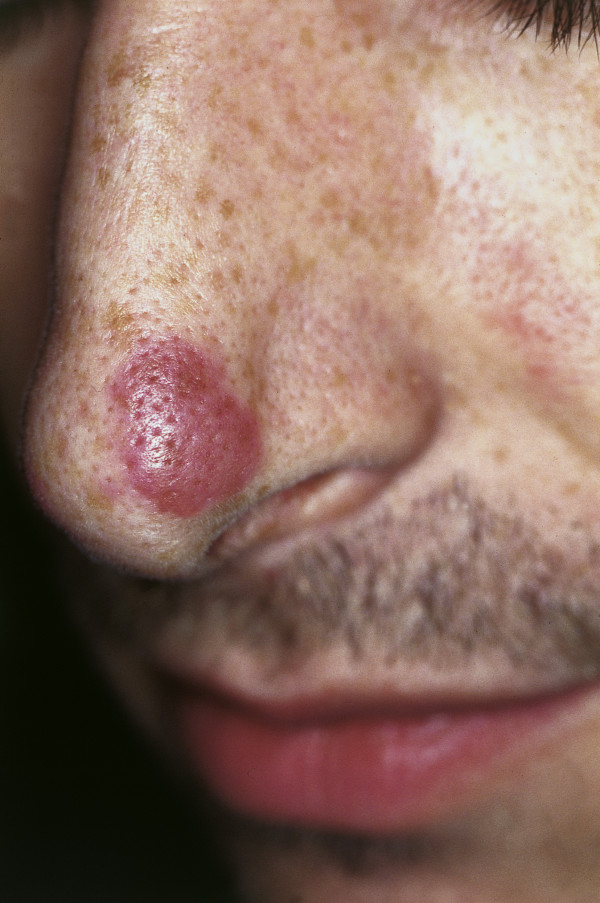
**Facial eosinophilic granuloma**. Red-brown nodule on the nose. Clearly visible follicular structures ("peau d'orange").

First described by Wigley in 1945, this condition is a chronic inflammation of the skin that generally occurs on the nose (Fig. 10), chin, forehead, temple or cheeks [101]. Clinically, round or oval brown-red macular and popular lesions with large follicular pores (giving the lesion an orange peel-like appearance) can be observed. Histologically, eosinophilia and patterns of leukocytoclastic vasculitis are characteristic. Therapy consists of dapsone p.o. (100-200 mg/day for four months) or intra-lesional steroid injections (e.g., triamcinolone 10 mg diluted with a local anesthetic 1:3-1:5). Dapsone therapy should be evaluated critically as the results are moderate, and the course of the disease is benign. Recently, the topical preparation of tacrolimus, a macrolide immunosuppressant, has been described as successful [102]. In cases of resistance to conservative therapy, the surgical excision of solitary lesions, cryotherapy, dermabrasion or ablative laser therapy (CO_2_, argon or erbium: YAG laser) should be considered.

### Sarcoidosis

**Figure 11:**
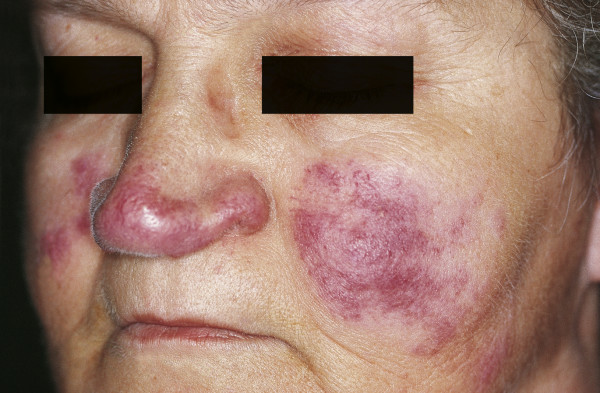
**Cutaneous lesions of sarcoidosis (lupus pernio)**. Red-to-purple indurated plaques and nodules affecting the nose and cheeks.

Sarcoidosis is a multisystem granulomatous inflammatory disease that can affect any organ. Cutaneous sarcoidosis is characterized by non-caseating granulomatas that consist of mononuclear phagocytes, epithelioid macrophages and multinucleate giant cells [103]. The macronodular type involving the nose and cheek is called lupus pernio and was first described by Besnier in 1889 [104]. The etiology of this disease is still unknown. Clinically, dark red, purple or violaceous plaques and nodules can be seen [Fig. 11]. The serum concentration of angiotensin-converting enzyme (ACE) is increased, and measurements have been used as an index of disease activity. Aside from topical and intra-lesional steroids, multiple forms of internal therapy (immunosuppressants such as steroids, interleukin-2 inhibitors or anti-tumor necrosis factor alpha treatment) have been described [105]. Pulsed dye or CO_2 _laser ablation is available for the debulking of granulomatous lesions; however, there are no evidence-based recommendations because of the limited number of patients treated [105].

## Pre-malignant tumors of the nose

### Actinic keratoses (syn: solar keratosis, senile keratosis)

Located on the nose, face, scalp, forearms and back of the hand, this very common pre-malignant lesion consists of crusty, scaly patches of skin. Size ranges from 2 - 10 mm, and colors such as pink, red or the same degree of pigmentation as the surrounding skin are observed. Actinic keratoses are associated with UV light exposure and therefore accompanied by solar damage to the surrounding skin. Patients are in or past middle age, very often with fair complexion. Histologically, five types can be distinguished: hypertrophic, atrophic, bowenoid, acantholytic and pigmented [106]. Left untreated this lesion can potentially result in squamous cell carcinoma. Approximately 20% of untreated actinic keratoses result in squamous cell carcinoma [107]. Therapy consists of simple curettage, topical photodynamic therapy, topical imiquimod, topical 3% diclofenac gel or 5-fluorouracil-creme. In case of surgical excision, histologic examination should be performed to exclude squamous cell carcinoma.

### Keratoacanthoma (syn.: molluscum sebaceum, molluscum pseudocarcinomatosum, idiopathic cutaneous pseudoepitheliomatous hyperplasia)

First described in 1889 by Hutchinson as a "crateriform ulcer of the face", keratoacanthoma is a fast-growing, epithelial tumor that develops from hair follicles or the surface epithelium of the skin. It can occur solitarily (frequent) or with multiple lesions (rare). The lesion consists of a firm, cone-shaped nodule (1-3 cm in diameter) with a central horn-filled crater. It shows rapid growth within weeks or months followed by spontaneous resolution over 4-6 months in most cases. Histologically and clinically it often resembles SCC. There is debate about whether it undergoes transformation into SCC or is SCC from the beginning [108,109]. Nevertheless, as SCC can masquerade as keratoacanthoma, surgical excision with an excision margin of 2-3 mm is recommended [106]. Because the histologic changes at the base of the lesion are important for histologic differentiation, a shave biopsy should be avoided and an excision of the lesion in its entirety should be performed [110]. Immunocompromised patients and those with Muir-Torre syndrome (the combined occurrence of at least one sebaceous skin tumor and one internal malignancy in the same patient) show an increased incidence of keratoacanthoma [111,112].

## Malignant tumors of the nose

The skin of the nose is a very common location for malignant tumors. UV-light exposure is a potent carcinogen of the skin, which results in frequent tumor involvement of the skin of the nose. In the following paragraph we present the most frequent malignant skin tumors of the nose.

### Melanoma

**Figure 12:**
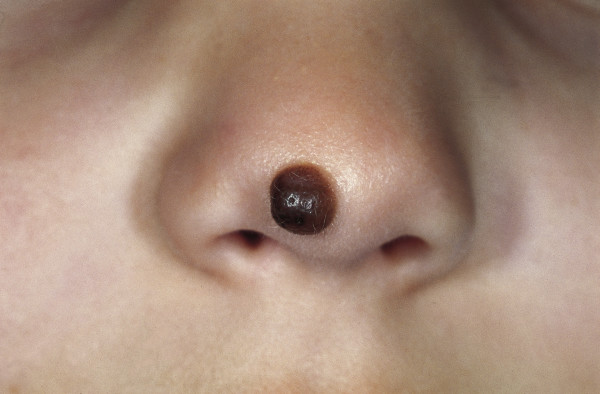
**Congenital melanocytic nevus**. Brown papule on the nose, which developed shortly after birth. The brownish exophytic lesion is well circumscribed.

Melanoma is the most devastating skin cancer with the highest increase in incidence in recent years, according to the World Health Organization (WHO). It has been estimated that incidences of melanoma will double every 10-20 years [113,114]. Melanoma originates from a malignant degenerated melanocyte, which is a highly aggressive tumor cell with poor rates of survival once it has metastasized. It can either develop *de novo *(70%) or from pre-existing melanocytic nevi (30%) (Fig. 12).

Unfortunately, there are only a few studies dealing specifically with melanoma on the nose. Jahn et al. have published the largest series of malignant melanomas on the nose so far [115]. In their group of 45 patients, they showed a female predisposition of 64.4%, with lentigo maligna melanoma (LMM) being the most frequent subtype (73.3%). In another study by Fisher et al., 36 patients with melanomas of the nose were described, whereas superficially spreading melanomas were reported in 47% and LMM in 25% of cases [116]. Forty-five percent of these cases were observed in female patients.

Therapy involves surgical excision by cold steel, similar to the procedure performed for cutaneous melanomas at other locations on the body. The recommended standard excision margins published by the American Cancer Society (ACS) and the German Association of Dermato-oncology (ADO) for melanoma of the skin are 10 mm for tumor thickness ≤ 2.00 mm and 20 mm for tumor thickness > 2.00 mm [117,118]. However, according to the ADO's guideline, in special localizations such as the facial, acral or anogenital regions a reduction of these margins is possible on the condition that micrographic controlled surgery is performed. However, current randomized trial evidence has recently shown to be insufficient in addressing optimal excision margins for primary cutaneous melanomas [119].

**Figure 13:**
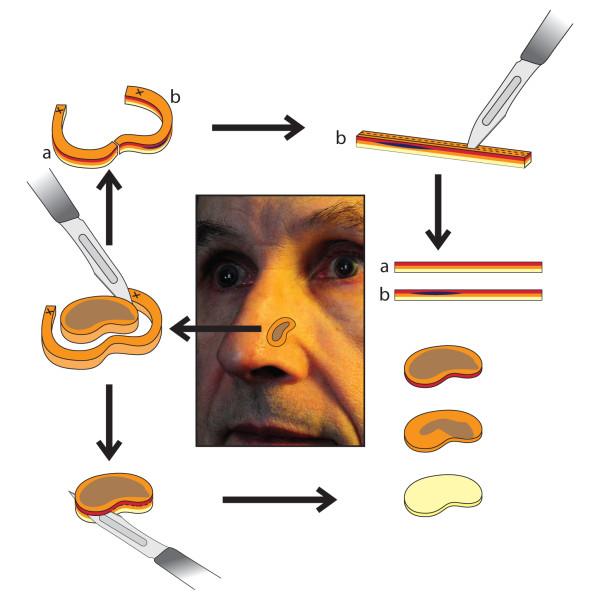
**Micrographic surgery according to the Tuebingen cake technique**. The base and the margin of the tumor are assessed separately. (modified according to Prof. Breuninger (120))

Although the nose has a distinct concave and convex anatomy, pre-operative tumor thickness can be assessed by ultrasound of the skin, depending on the localization of the melanoma [120,121]. In cases of LMM, different techniques of 3D histology have been described. Some authors prefer the Tuebingen cake technique, whereas other authors prefer classic Mohs surgery [122-124]. Micrographic surgery according to the Tuebingen cake technique has been studied by Jahn and colleagues [115]. It ideally utilizes a cylindrical piece of tissue where the base and the margin of the tumor are assessed separately (Fig. 13).

**Figure 14:**
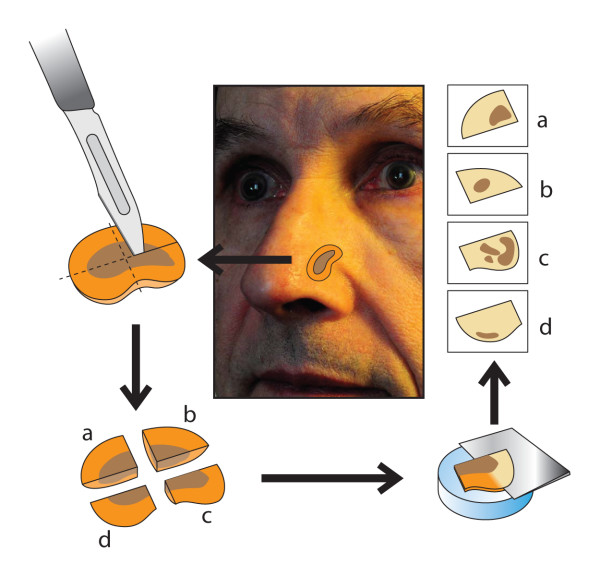
**Mohs surgery allowing the complete circumferential peripheral and deep margin assessment, using frozen section histology**. (modified according to Prof. Breuninger (120))

Mohs surgery allows complete circumferential peripheral and deep margin assessment using frozen section histology. In classic Mohs surgery, the tissue is excised in a cone-shaped pattern with a very small surgical margin (1 to 1.5 mm of visually uninvolved skin). Specimen preparation consists of cutting the specimen on the cryostat, placing sections on slides, followed by staining and evaluation by the Mohs surgeon (Fig. 14). The special method of tissue processing and staining in Mohs surgery has been compared with peeling an orange, where the peel is the surgical margin that is removed and flattened out for further examination [125]. Actually there are no equivalent data to compare both methods.

Jahn et al. conclude from their study data that male patients tend to have fewer recurrences than female patients and that LMM has a better prognosis than other histologic subtypes in patients with stage I and II melanoma of the nose [115]. The authors report recurrence rates of 6.7% with all recurrences observed in female patients. The prognoses with stage I and II melanoma of the nose were good, with a survival rate of 97.8% over three years and 95.6% over five years. Unfortunately, there were no data available for patients with stage III melanoma. Jahn et al. further conclude that although tumor thickness is the most important prognostic factor for cutaneous melanoma of the nose, this factor has no significant influence on the prognosis, probably because of the limited number of patients (n = 45). To date all available studies on elective lymph node dissection (ELND) have failed to demonstrate a beneficial effect on patients with cutaneous melanoma of the trunk and extremities; therefore, there is limited evidence to support application of this technique in patients with melanoma of the nose [115,126-128]. Although a sentinel lymph node biopsy (SLNB) is performed in cutaneous melanomas of other localizations with a tumor thickness > 1.00 mm, the available data for patients with melanomas of the nose do not suggest a clear recommendation regarding prognostic impact. In contrast to the relatively good prognosis for stage I and II melanomas of the skin of the nose, melanoma with sinonasal involvement arising from the nasal cavity and paranasal sinuses is associated with generally poor survival rates [129]. A high rate of local recurrence (31-85%), common distant metastasis (25-50%) and a poor five-year survival rate (13-45%) all make this form of nasal melanoma the most lethal [130-134].

### Basal cell carcinoma (Syn.: basalioma, basal cell epithelioma)

Basal cell carcinoma (BCC) is the most common malignancy in humans and accounts for more than 90% of all malignant cutaneous lesions of the head and neck [133]. Because UV light associated with chronic sun exposure is the main risk factor, BCC commonly occurs on the face, with the nose being the most frequently affected location and the alae, dorsum and tip being the parts most frequently affected [134].

**Figure 15:**
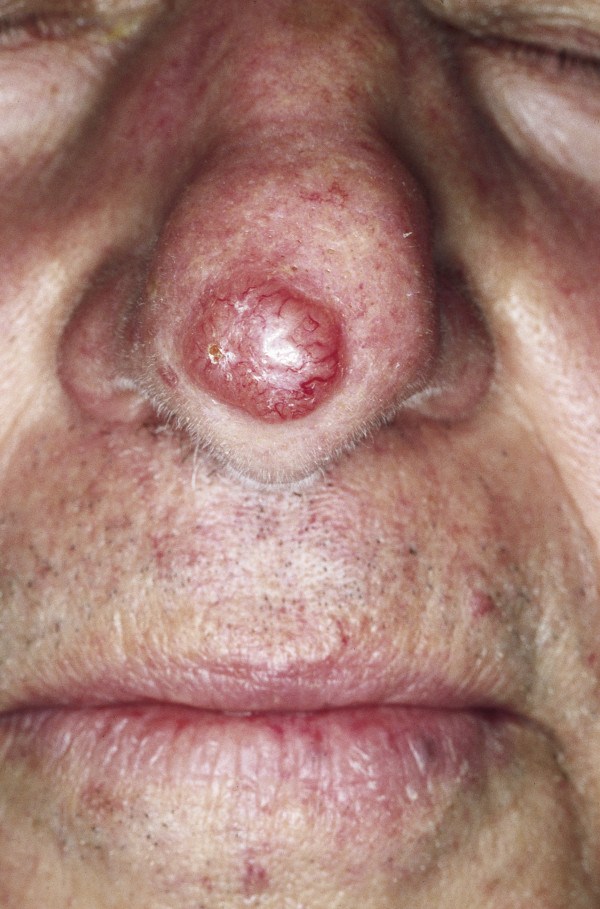
**BCC Nodular type**. Red, waxy nodule on the tip of the nose. Visible telangiectasias over the surface.

**Figure 16:**
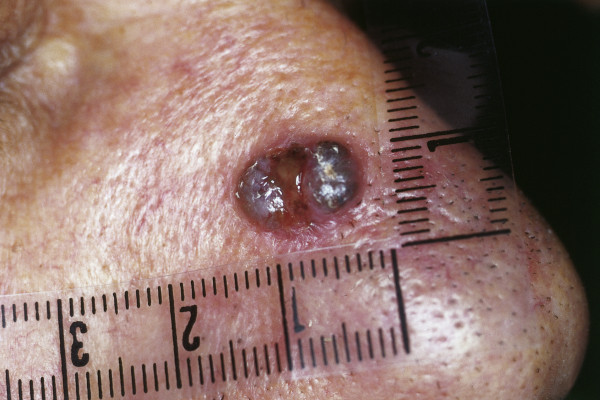
**Pigmented BCC**. Dark nodule (resulting from melanin deposition) at the alar of the nose. Small ulceration at the center.

**Figure 17:**
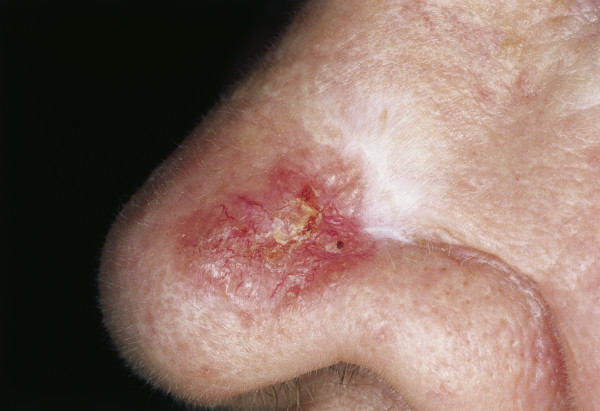
**Scar-like morphea-like BCC**. Sclerotic, partially reddish plaque. Crusting in the center.

Although it rarely metastasizes, untreated BCC can cause considerable disfigurement and is potentially life threatening when eroding vital structures. Five BCC subtypes with different clinical behavior can be distinguished: pigmented, cystic, superficial multicentric, morphea-like and nodular-ulcerative types (with the last being the most common, Fig. 15). Pigmented BCC can mimic melanoma upon clinical examination and usually occurs in sun-exposed areas [Fig. 16] [135]. Morphea-like BCC shows a lingular growth pattern and varies in size [Fig. 17]. It is a rare morphological variant of BCC (roughly 2% of all BCCs) and is the most insidious form because the degree of infiltration can far exceed what is clinically visible, because the tumor grows in an 'iceberg'-like pattern with only the top of the tumor visible [136,137].

A variety of different treatment options such as cryotherapy, photodynamic therapy, application of imiquimod or 5-fluourouracil, electrodessication and radiation therapy have been described. However, micrographic-controlled surgery is the gold standard with the lowest rate of recurrence (1.0-5,6%) [138-144]. The nose, which is part of the so called *H-zone *of the face, shows the highest rate of recurrence compared with other localizations [145]. Embryonic fusion planes such as the nasolabial fold or the medial canthus can be affected by large BCCs of the nose, possibly contributing to tumor recurrence.

### Squamous cell carcinoma (syn.: spinalioma)

Cutaneous squamous cell carcinoma (SCC) accounts for approximately 10% of skin malignancies on the nose. It is more common in men and 70% of cases are located in the head and neck area [146]. It is related to chronic sun exposure and immunosuppression and rarely arises from normal-appearing skin. SCC typically develops on sun-damaged skin or actinic keratoses and less frequently on scars from burns [147,148]. In patients having undergone renal transplants and immunosuppression, the incidence has been 18 times greater than in healthy individuals [149]. Clinically, SCC presents as an erythematous crusting, sometimes ulcerated, lesion with a red granular base. It shows a tendency to bleed with minimal trauma. The diagnosis and extent of the lesion sometimes necessitate multiple biopsies. When SCC arises in sun-damaged skin, a minority of patients develop metastases (0.5%) [150]. However, in all patients with SCC of the skin, the metastasis is more frequent (2-3%), and most cases are located in the cervical lymph nodes or parotids [151,152]. The likelihood of metastasis increases with tumors with a diameter of at least 15 mm and a Breslow tumor thickness (vertical) of at least 2 mm [137]. Death occurs in three-quarters of patients with metastasis [153,154]. The parotid gland is the "metastatic basin" for cutaneous SCC of the head and neck because it drains via lymphatic vessels on the nose, cheek and forehead [155]. In cases of parotid involvement, a parotidectomy with or without a simultaneous neck dissection is the procedure of choice. Clark levels IV or V are associated with a 20% regional metastatic rate. *De novo *lesions, an increased depth of invasion (beyond 4-5 mm), tumor size (> 2 cm) and desmoplastic SCCs are associated with a higher rate of metastasis. The same is true for adenoid and mucin-producing types, SCCs of the lower lip (metastatic rate 16%), SCCs on burn scars (18%), radiation-induced SCCs (20%) and/or osteomyelitic sinuses (31%) [137,156-160].

Micrographic-controlled surgery is the treatment of choice. Excision margins of 4 mm and 6 mm have been suggested for lesions less than and greater than 2 cm, respectively [160]. Because there are no large randomized studies regarding excision margins for cutaneous SCCs, these are rough guidelines. The surgeon's experience and judgment in planning surgical treatment is therefore significant for successful treatment [160]. In cases where patients are unable to undergo surgery radiation, therapy has been described as successful with cure rates similar to those obtained with standard surgical excision. Although chemotherapy has not been effective, some studies report that epidermal growth factor receptor (EGFR) inhibitors might be useful adjuncts to surgical treatment [161,162].

### Kaposi's sarcoma (KS)

KS was first described in 1872 by the Hungarian dermatologist Moritz Kaposi and is a carcinoma arising from the endothelial lining of lymphatic tissue [163]. The histology is characteristic and shows an excessive proliferation of spindle cells, slit-like vascular spaces and extravasated erythrocytes. Principally, KS can arise anywhere on the skin or mucosa of the body, including internal organs. The lower extremities of the skin (especially the soles of the feet) and the head and neck are typically involved.

**Figure 18:**
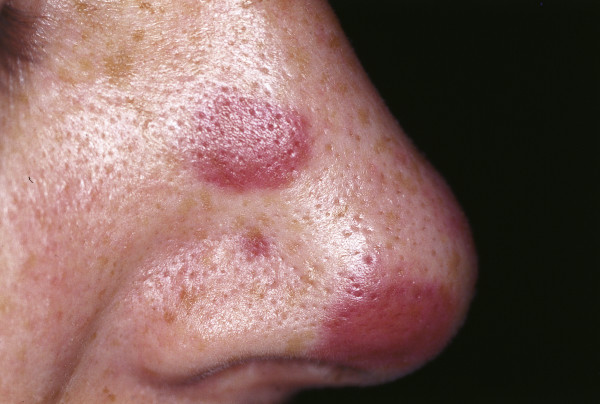
**Kaposi's sarcoma**. Characteristic violaceous plaques on the alar and tip of the nose in an HIV-positive female patient.

Masih et al. used bronchoscopy to evaluate 19 HIV-positive patients with pulmonary KS [164]. Fifteen of these patients also had oral-facial KS and 13 showed a prominent tip-of-the-nose KS lesion. The authors concluded that tip-of-the-nose KS lesions are commonly associated with pulmonary KS and should be noticed as a sentinel sign for pulmonary KS, suggesting that bronchoscopy should be considered for these patients. On clinical examination the vascular pattern results in a dark red to blue or violaceous appearance, as the vascular spaces within the lesions fill with blood. The lesions are non-pruritic and appear as macular (Fig. 18), papular, nodular or plaque-like. Four different types have been distinguished in the literature. The classic type mainly occurs in Mediterranean men (male-to-female ratio of 10-15:1) of 50-70 years of age [165,166]. The endemic African type occurs in HIV-negative individuals and shows a tendency for lymph node involvement. The immunocompromised type can occur in individuals just after organ transplantation [167,168]. Finally, the AIDS-related type is now the most commonly presented. It is seen in patients with advanced HIV infection or no access to highly active antiretroviral therapy (HAART). It is the most common malignancy seen in HIV-infected patients [169,170].

Over 90% of lesions, regardless of the KS type, are associated with DNA virus human herpes virus 8, also called KS-associated herpesvirus (HHV-8 or KSHV), which has been identified as the primary trigger [171]. Concerning therapy, a variety of modalities have been described. Therapeutic options include systemic therapy in HIV-positive patients (HAART), systemic chemotherapy with doxorubicin, conventional radiation therapy, electron beam radiation therapy (EBRT), surgical excision, topical retinoids, cryotherapy, laser therapy and intra-lesional therapy with vincristin, vinblastin or bleomycin [172,173].

## Conclusion

The most important skin diseases of the nose, which might require surgical consultation or laser therapy, have been described briefly in this review. In conclusion, the authors suggest that all disciplines that offer conservative or surgical treatment must be familiar with the special morphology and characteristics of skin diseases of the nose. In the case of complex lesions an interdisciplinary approach that combines dermatology, otolaryngology and surgery can provide optimal care for the patient.

## Consent

Written informed consent was obtained from the patients/guardians of the patient for publication of this review article and accompanying images. A copy of the written consent is available for review by the Editor-in-Chief of this journal.

## Competing interests

The authors declare that they have no competing interests.

## Authors' contributions

MS: Documented and prepared the draft. DS: Edited the manuscript, revised the bibliography and helped prepare the draft. CT: Searched the literature and revised and edited the manuscript. VP: Searched the literature, photography and helped edit the manuscript. PA: Revised the manuscript, searched the literature and helped edit the manuscript. FGB: Helped prepare the draft and edited most of the manuscript. All authors read and approved the final manuscript.
